# Psychological Aspects and Quality of Life in Chronic Pain

**DOI:** 10.1155/2019/8346161

**Published:** 2019-06-12

**Authors:** Panagiotis Zis, Giustino Varrassi, Athina Vadalouka, Antonella Paladini

**Affiliations:** ^1^Medical School, University of Cyprus, Nicosia, Cyprus; ^2^Paolo Procacci Foundation, Via Tacito 7, Roma, Italy; ^3^Athens Medical Centre, Athens, Greece; ^4^Department of MESVA, University of L'Aquila, L'Aquila, Italy

Pain, if not the worst, is one of the worst symptoms that a patient experiences and has a detrimental effect on the patients' quality of life [1]. Chronic pain, usually defined as lasting for a period of more than 3 months, is prevalent in a variety of diseases including peripheral neuropathy [2–8], neurodegenerative diseases [9, 10], and cancer [11]. Presence of chronic pain has an independent additional burden to patients' mental health, increasing significantly the risk of depression [12]. Successful management of pain, with both pharmacological and nonpharmacological approaches, can improve the quality of life and ameliorate patients' mental health status [13–18].

In this special issue, the readers will find ten articles, covering a wide spectrum of types of pain and types of interventions and comprising “Association of depression/anxiety symptoms with neck pain: a systematic review and meta-analysis of literature in China” by F. Liu et al.; “Pain intensity is not always associated with poorer health status: exploring the moderating role of spouse personality” by C. Suso-Ribera et al.; “Topical review: basic psychological needs in adolescents with chronic pain—a self-determination perspective” by A. Riggenbach et al.; “The mediating effect of central sensitization on the relation between pain intensity and psychological factors: a cross-sectional study with mediation analysis” by H. Shigetoh et al.; “Negative affect, type D personality, quality of life, and dysfunctional outcomes of total knee arthroplasty” by M. Vogel et al.; “Pain acceptance and its associated factors among cancer patients in mainland China: a cross-sectional study” by X. Xu et al.; “Reward processing under chronic pain from the perspective of “liking” and “wanting”: a narrative review” by X. Liu et al.; “Living with chronic pain: a qualitative study of the daily life of older people with chronic pain in Chile” by I. Rodríguez et al.; “Personality and personality disorders in medication overuse headache: a controlled study by SWAP-200” by F. Galli et al.; and “Quality of life in painful peripheral neuropathies: a systematic review” by A. Girach et al.

## References

[1] Zis P., Sarrigiannis P. G., Rao D. G., Hadjivassiliou M. (2018). Quality of life in patients with gluten neuropathy: a case-controlled study. *Nutrients*.

[2] Zis P., Paladini A., Piroli A., McHugh P. C., Varrassi G., Hadjivassiliou M. (2017). Pain as a first manifestation of paraneoplastic Neuropathies: a systematic review and meta-analysis. *Pain and Therapy*.

[3] Brozou V., Vadalouca A., Zis P. (2018). Pain in platin-induced neuropathies: a systematic review and meta-analysis. *Pain and Therapy*.

[4] Artemiadis A. K., Zis P. (2018). Neuropathic pain in acute and subacute Neuropathies: a systematic review. *Pain Physician*.

[5] Zis P., Sarrigiannis P. G., Rao D. G., Hadjivassiliou M. (2018). Gluten neuropathy: prevalence of neuropathic pain and the role of gluten-free diet. *Journal of Neurology*.

[6] Zis P., Grünewald R. A., Chaudhuri R. K., Hadjivassiliou M. (2017). Peripheral neuropathy in idiopathic Parkinson’s disease: a systematic review. *Journal of the Neurological Sciences*.

[7] Julian T., Glascow N., Syeed R., Zis P. (2018). Alcohol-related peripheral neuropathy: a systematic review and meta-analysis. *Journal of Neurology*.

[8] Zis P., Sarrigiannis P. G., Rao D. G., Hewamadduma C., Hadjivassiliou M. (2019). Chronic idiopathic axonal polyneuropathy: prevalence of pain and impact on quality of life. *Brain Behav*.

[9] Adewusi J. K., Hadjivassiliou M., Vinagre-Aragón A. (2018). Peripheral neuropathic pain in idiopathic Parkinson’s disease: prevalence and impact on quality of life; a case controlled study. *Journal of the Neurological Sciences*.

[10] Adewusi J. K., Hadjivassiliou M., Vinagre-Aragón A. (2018 Sep). Sensory neuropathic symptoms in idiopathic Parkinson’s disease: prevalence and impact on quality of life. *Acta Neurologica Belgica*.

[11] Zis P., Varrassi G. (2017). Painful peripheral neuropathy and cancer. *Pain and Therapy*.

[12] Zis P., Daskalaki A., Bountouni I., Sykioti P., Varrassi G., Paladini A. (2017). Depression and chronic pain in the elderly: links and management challenges. *Clinical Interventions in Aging*.

[13] Vadalouca A., Raptis E., Moka E., Zis P., Sykioti P., Siafaka I. (2012). Pharmacological treatment of neuropathic cancer pain: a comprehensive review of the current literature. *Pain Practice*.

[14] Stavropoulou E., Argyra E., Zis P., Vadalouca A., Siafaka I. (2014). The effect of intravenous lidocaine on trigeminal neuralgia: a randomized double blind placebo controlled trial. *ISRN Pain*.

[15] Zis P., Apsokardos A., Isaia C., Sykioti P., Vadalouca A. (2014 Mar-Apr). Posttraumatic and postsurgical neuropathic pain responsive to treatment with capsaicin 8% topical patch. *Pain Physician*.

[16] Varrassi G., Fusco M., Skaper S. D. (2018). A pharmacological rationale to reduce the incidence of opioid induced tolerance and hyperalgesia: a review. *Pain and Therapy*.

[17] Zis P., Bernali N., Argira E., Siafaka I., Vadalouka A. (2016). Effectiveness and impact of capsaicin 8% patch on quality of life in patients with lumbosacral pain: an open-label study. *Pain Physician*.

[18] Vitoula K., Venneri A., Varrassi G. (2018). Behavioral therapy approaches for the management of low back pain: an up-to-date systematic review. *Pain and Therapy*.

